# The influence of water content on the longitudinal modulus of elasticity of maize stalk pith and rind tissues

**DOI:** 10.1186/s13007-023-01039-5

**Published:** 2023-06-30

**Authors:** Brandon Sutherland, Kirsten Steele, Joseph Carter, Douglas D. Cook

**Affiliations:** Department of Mechanical Engineering, Brigham Young University, Provo, UT 84602 USA

**Keywords:** Modulus of elasticity, Maize, Pith, Rind, Water content

## Abstract

**Background:**

Modern computational modeling could provide the key to obtaining new insights into the mechanisms of maize stalk failure as well as suggesting new ways to improve stalk strength. However, a complete set of mechanical properties of maize tissues is required to enable computational modeling of maize stems. This study developed two compression test methods for obtaining the longitudinal modulus of elasticity of both rind and pith tissues, assessed the influence of water content on tissue properties, and investigated the relationship between rind modulus and pith modulus. These methods involved uniform 5–7 cm segments of maize stems which were scanned using a flatbed scanner then tested in compression using a universal testing machine in both intact and dissected (rind-only and pith-only) states.

**Results:**

The modulus of elasticity of pith tissues was highest for fully turgid specimens and decreased as water was removed from the specimens. Water content was negatively correlated with the modulus of elasticity of the rind. Rind and pith tissues were found to be weakly correlated. The median ratio of rind modulus to pith modulus was found to be 17. Of the two methods investigated, the pith-only specimen preparation was found to be simple reliable while the rind-only method was found to be adversely affected by lateral bowing of the specimen.

**Conclusions:**

Researchers can use the information in this paper to improve computational models of maize stems in three ways: (1) by incorporating realistic values of the longitudinal modulus of elasticity of pith and rind tissues; (2) by selecting pith and rind properties that match empirically observed ratios; and (3) by incorporating appropriate dependencies between these material properties and water content. From an experimental perspective, the intact/pith-only experimental method outlined in this paper is simpler than previously reported methods and provides reliable estimates of both pith and rind modulus of elasticity values. Further research using this measurement method is recommended to more clearly understand the influence of water content and turgor pressure on tissue properties.

**Supplementary Information:**

The online version contains supplementary material available at 10.1186/s13007-023-01039-5.

## Introduction

Maize stalk failure (lodging) is a major problem for farmers and has proven to be a challenging problem for plant scientists for over 100 years [11]. In recent years, computational modeling has begun to be used to address this problem [33]. The in silico approach is very promising as it enables control over critical variables that cannot be controlled in an experimental setting.

Like many other plant stems, maize utilizes a structural architecture consisting of a tough outer rind filled by a foam-like interior (pith). While this architecture is also observed elsewhere in nature (bone, bird feathers, etc.), plant stems are capable of *regulating* turgor pressure, which affects mechanical tissue properties [22]. When water content and turgor pressure are high, plant tissues exhibit a higher modulus of elasticity [10, 18, 32]. Conversely, when the cells are flaccid, the modulus of elasticity is reduced. Since stiffness and strength are often closely related, plants are capable of actively regulating the mechanical properties of their tissues [19].

When applying modern computational tools to maize and other plants, a common challenge is the relatively limited data on the mechanical properties of stem tissues. Maize stalk tissues are well-approximated as transversely isotropic [27, 29, 33]. The behaviors of transversely isotropic materials are governed by five mechanical properties [7]. These include two modulus of elasticity properties, one each in the axial and transverse directions, two shear modulus properties, one each in the axial and transverse directions, and the longitudinal Poisson’s ratio. When two tissue types (rind and pith) are used to describe the behavior of maize tissues, a total of 10 material properties are required to complete the model.

Models are highly beneficial because they enable absolute control of model conditions and behavior. While models are only approximations of reality, the increased level of control provided by a model enables examination of aspects of behavior that are not possible using experiments alone. As a complete set of properties are not yet present in the research literature, computational models are currently built using *estimates* of the missing properties [27, 29, 33]. This obviously influences the results of simulations. The primary purpose of this study is to provide measurements of maize pith and rind tissues as functions of water so that future modeling studies can be based more closely on measured data with less reliance on estimates of unknown tissue properties.

The longitudinal modulus of the rind is the most important material property for modeling maize stalks because it bears the vast majority of bending, axial, and torsional loads [24]. Hence, several studies have been carried out to measure this property [1, 2, 35]. The transverse modulus of the pith and rind have been assessed using inverse finite-element models [30] and x-ray computed tomography has been used along with finite-element models to obtain estimates of the distribution of transverse modulus of elasticity values [28]. One prior study has reported the longitudinal modulus of elasticity and ultimate strength of maize pith tissue as well as the effect of moisture content [36]. However, that study used dogbone-shaped specimens which are not appropriate for testing fibrous materials [5]. In addition, Zhang et al., reported that modulus of elasticity was negatively correlated with moisture content, a finding which is contradicted by numerous studies that have shown a positive correlation between parenchyma tissue and moisture content (see following paragraph). While shear toughness of maize stems has been investigated [9, 25], the authors are unaware of any studies that have measured the shear modulus of the rind or pith, or the Poisson’s ratio of maize tissues. Clearly, more research is needed on the material properties of maize, as it is (by weight) the world’s top crop.

The mechanical properties of plant tissues are known to be influenced by water content, but water content affects different tissue types in different ways. In general, the modulus of elasticity of parenchyma tissue typically increases as water content increases. This relationship has been demonstrated in numerous studies involving the parenchyma of pumpkin, potato. apple, banana, and carrot [16–18, 23, 26]. In contrast, the modulus of elasticity of sclerenchyma tissue decreases as water content increases. This relationship is well known in wood, bamboo, and has been demonstrated in leaves of wheat and rhododendron [6, 12, 22, 34].

The overall purpose of this study was to obtain measurements of maize pith and rind tissues as functions of water content in support of future modeling activities. Specific objectives were to (a) develop simplified methods for assessing the longitudinal modulus of elasticity values of maize pith and rind tissues; (b) measure pith and rind tissues as functions of water content; and (c) investigate the relationship (if any) between these properties. Such information is needed to fully enable future computational modeling studies that are able to accurately simulate maize stalks behavior.

## Methods

### Overview of the testing process

**Fig. 1:**
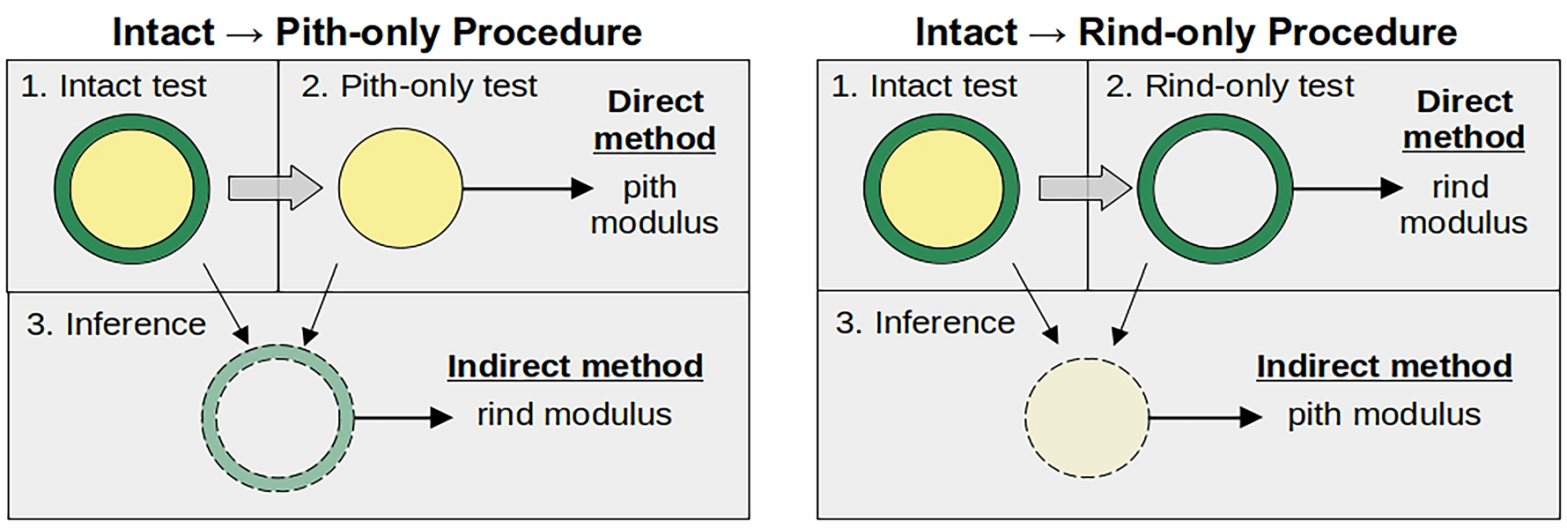
Cross-sectional illustration of the types of specimens used in compression tests. The top row of each panel illustrates a specimen tested intact, then dissected. The dashed line specimens indicate tissues which were destroyed during dissection, but for which the modulus was inferred using both tests

In this study, a series of compression tests were used to estimate the material properties of tissues with varying levels of water content. All test specimens were taken from maize stalk internodes. Only internodes exhibiting a nearly uniform cross-sectional shape were chosen for testing. Specimens were 6–8 cm in length. Three types of specimens were tested: intact specimens (no dissection); rind-only specimens (pith removed); and pith-only specimens (rind removed). All specimens were initially tested in the intact state. Next, either rind or pith tissue was carefully removed to create rind-only or pith-only specimens. Each rind-only or pith-only specimen was then tested over the course of 24 h to assess the changes in elastic modulus for each tissue type as a function of water content. Rind-only and pith-only specimens were used to directly assess elastic modulus. In addition, the modulus of removed tissue was inferred by combining intact test results with direct measurement test results. Throughout the paper, these two approaches are referred to as “direct” and “indirect” methods. The types of specimens are shown graphically in Fig. 1.

### Stalk samples

Maize stalks were grown in an open field during the 2020 growing season. Varieties included two types of dent maize (Vigor Root, Extra Early), sweet corn (Silver Queen), and flint corn (Fiesta Ornamental). Stalks were harvested once the maize plants were near or at full physiological maturity. The stalks were cut with pruning shears just above the root and immediately transferred to the lab for specimen preparation.

### Sample preparation

#### Intact specimens

Intact specimens were selected from internode regions having a straight, uniform appearance. Prior to testing, specimens were cut into segments with a minimum length-to-diameter ratio of at least 3 (ASTM-D4761,). Two self-leveling laser levels were used to ensure the proper alignment of the specimen and the platens. The laser levels were placed such that they projected perpendicularly to each other and projected down the center of the platen. The samples were then placed between two self-aligning platens such that both lasers ran parallel and down the center of the sample.

#### Water content and water loss calculations

After initial testing (intact state and rind-only or pith-only tests), each specimen was left in open air in a room temperature environment in which the water content slowly decreased. Specimens were re-tested at time intervals of 4, 8, and 24 h after the initial testing. Once all tests were completed, the samples were dried in a dehydrator at 50 °C for 12 h. The drying process tended to cause geometric distortion (warping) of specimens. As a result, mechanical tests were not performed on dried specimens.

Relative water content at each time point was calculated using the current specimen mass (*M*_*i*_), the specimen dry mass (*M*_*d*_) as follows:

1 $$Water\,Content \left(\%\right) =\frac{{M}_{i}-{M}_{d}}{{M}_{d}}*100$$

While useful, the above formulation is somewhat problematic for longitudinal comparisons because the denominator changes over time. Water Content ends at 0% for all dry specimens, but each turgid specimen begins at a unique value of Water Content that is not equal to 100%.

Water content can also be quantified as relative water loss: the percent water remaining in each specimen relative to the original water mass in the specimen. With *M*_*0*_ indicating the initial sample mass, relative water loss is calculated as:

2 $$Water\,Loss \left(\%\right) =\frac{{M}_{0} - {M}_{i}}{{M}_{0} - {M}_{d}}*100$$

Water Loss facilitates comparisons between specimens because it begins at 0% for each specimen, and increases to 100% for all dry specimens. In addition, water loss was found to provide greater insight into the influence of water on tissue properties than water content.

#### Testing procedure

Testing involved a cyclic pattern. The specimen was loaded to a maximum compressive force of either 100N or 150N (depending on the size of the specimen) at 10 mm/min before being returned to a compression of 50N and reloaded again to 100N or 150N. This compression cycle was repeated 6 times to ensure adequate preconditioning [1]. Load and displacement data was logged simultaneously at 200 Hz. Only data from the last testing cycle was reported. Previous studies involving compression tests of maize stalks used deflectometers [1, 2]. Deflectometers were not used in this test protocol because they were found to cause damage to both the rind-only or pith-only specimens.

### Data processing

#### Direct method for measuring modulus of elasticity

The modulus of elasticity relates the stress and strain of a material. For uniaxial stress, the modulus of elasticity (E_i_) of single-tissue specimens was computed by using the force/deformation slope (*F*_*i*_/δ_*i*_), as well as the length and cross-sectional area of the specimen (*L*_*i*_ and *A*_*i*_, respectively):

3 $$E_{i} = \frac{{F_{i} \;L_{i} }}{{\delta_{i} \;A_{i} }}$$

In the results section, pith-only modulus of elasticity is notated as *E*_*p*_ and the modulus of the rind is notated as *E*_*r*_.

#### Indirect method for measuring modulus of elasticity

One purpose of this study was to investigate relationships between the modulus of elasticity of the rind and the pith. However, the dissection process always required the destruction of one tissue type and comparisons of the modulus of elasticity of rind and pith across specimens cannot be used to examine the relationship between the properties. Fortunately, the modulus of the destroyed tissue can be inferred through the use of data from two different tests. This requires three elements: (1) the known modulus of one tissue as explained above; (2) measurements from the intact specimen; and (3) an equation describing the mechanical response of the two-tissue specimen.

Assuming uniaxial compression, the relationship of Eq. 3 can be rearranged in the form of a simple linear spring (*F* = *kx*), where* x* is the deformation (*δ*) of the sample and the stiffness of the sample is captured by the term *EA/L*:

4 $$F = \frac{EA}{L}\delta$$

When two materials are placed in compression together (such as the rind and pith of an intact specimen) this relationship becomes:

5 $$F_{agg} = \left( {E_{i} A_{i} + E_{j} A_{j} } \right)\frac{{\delta_{agg} }}{L}$$

Assuming that *E*_*i*_ was found using a dedicated rind-only or pith-only test (Eq. 3), we can solve for the remaining modulus value as:

6 $$E{}_{j} = \frac{{F_{agg} L}}{{\delta_{agg} A_{j} }} - E_{i} \frac{{A_{i} }}{{A_{j} }}$$

Here the “*agg*” subscript stands for aggregate and refers to measurements from the intact-specimen test. The areas, *A*_*i*_ and *A*_*j*_ are measured from the intact specimen, and *L* is the same specimen length used in Eq. 3.

Material properties can only be inferred for situations where both intact and dissected specimen tests are available. In this study, this was the case for only fully turgid specimens. In other words, both properties were available for turgid specimens, but only one property was available as the drying processes progressed.

## Results

### Modulus of elasticity values for turgid specimens

**Fig. 2:**
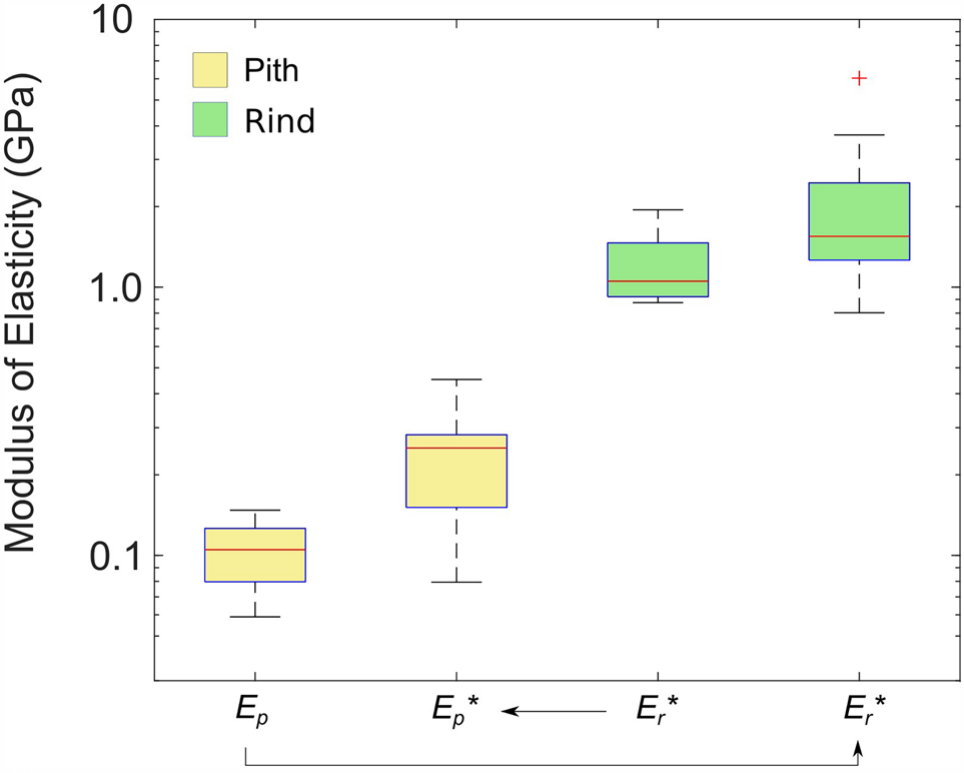
Modulus of elasticity values for fully turgid rind and pith tissues. Arrows point from directly measured specimens to the corresponding data for inferred properties

The distributions of longitudinal modulus values for turgid specimens are shown in Fig. 2. The obtained values are broken down by calculation method (direct or inferred). As shown in this figure, measurements of rind modulus ranged from 0.8 to 3.7 GPa. The pith modulus ranged from 0.05 to 0.5 GPa. Arrows in the figure indicate the direction of inference (i.e. which properties were inferred, and from which set of direct measurements). The aggregate modulus of elasticity for turgid, intact stem specimens ranged from 0.2 to 1.4 GPa.

### Relationship between modulus values of turgid specimens

The data of Fig. 2 was used to compute the ratio of rind modulus to pith modulus for turgid specimens. This was done in two ways: first by using the data in which pith modulus was measured directly, and rind was inferred; and second, by using the data in which rind was measured directly and the pith modulus was inferred.

**Fig. 3:**
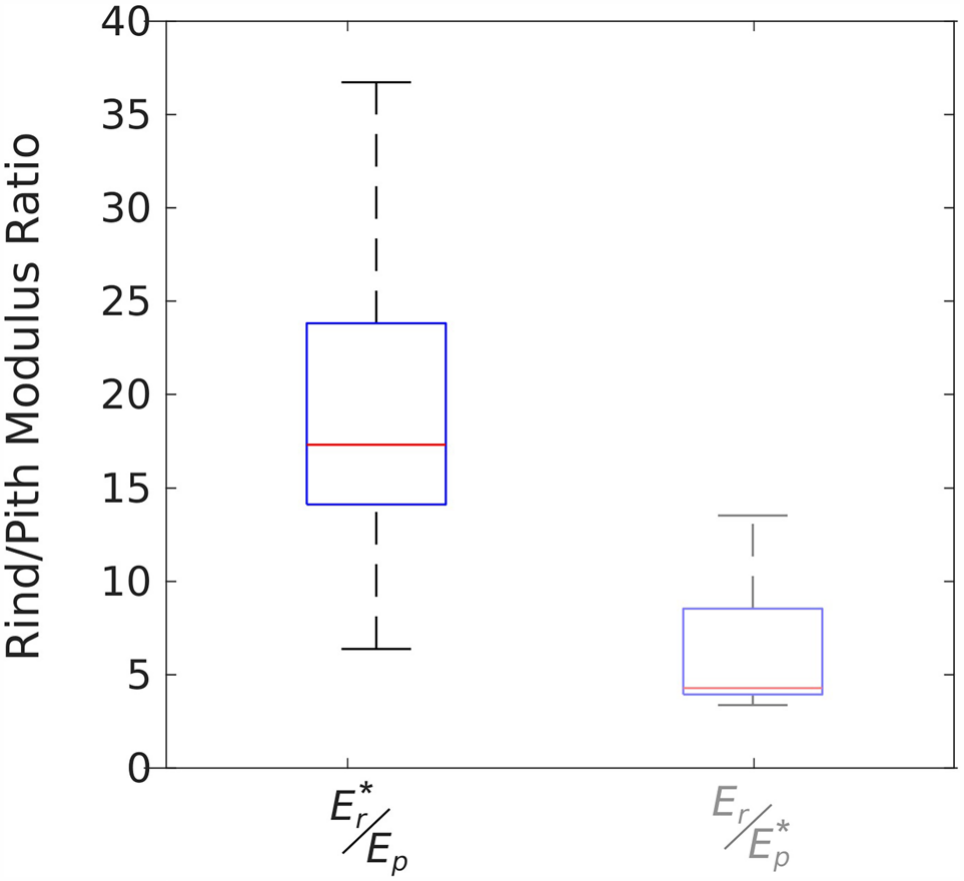
Distributions of rind:pith modulus of elasticity ratios for fully turgid specimens. The inferred modulus values are indicated by an asterisk. The box on the right is faded to draw attention to the fact that it involves ratios of less accurate values

As shown in Fig. 3, these two methods produced very different distributions of rind/pith ratios. The reason for this discrepancy appears to be related to the mechanics of compression testing. In compression test such as these, the modeling equations used (Eqs. 2, 3, 4, 5) assume that all tissue deformation is in the direction of the applied compressive load. Intact and pith-only specimens satisfied this assumption reasonably well because their aspect ratio (height:width ratio) ranged from 2.5 to 3.5. Lower ratios effectively prioritize axial compression over bending deformation. In contrast, rind-only specimens are essentially hollow cylinders. The height-to-thickness ratio of specimen walls was very high, ranging from 50 to 90. At such high ratios, bending becomes much more prevalent. Thus, hollow rind-only specimens are more prone to lateral deformation during compression tests (i.e., the rind can more easily deflect or “bow” perpendicular to the direction of loading). The supplementary material that accompanies this paper provides additional explanation of this phenomenon, supported by finite-element modeling results of maize stalk specimens (Additional File 1).

Lateral deformation acts to reduce the force/deformation slopes of rind-only specimens which in turn reduce the modulus of the rind. As seen in Fig. 2, the directly measured rind modulus values (*E*_*r*_) are lower than those obtained through inference (*E*_r_*). On the other hand, lateral deformation is less likely to occur in intact specimens because it is suppressed by the presence of the pith tissue [15]. With minimal lateral bowing of intact specimens, the inferred pith modulus (*E*_p_*) would be shifted *upwards* to compensate for the lower rind stiffness caused by lateral deformation of rind-only specimens. The net result is that rind-only specimens yielded an artificially low modulus, and the corresponding pith modulus values exhibited artificially high modulus values.

The effect of lateral bowing can be seen in both Fig. 2 (direct/indirect figure), and Fig. 3 (ratios figure). The pith-only test data should therefore be interpreted as being more accurate than the rind-only data, which should be interpreted as providing a lower bound on rind modulus values. For pith-only tests, the median rind/pith ratio was 17.3 with first and third quartiles at 14.1 and 23.8, respectively.

**Fig. 4:**
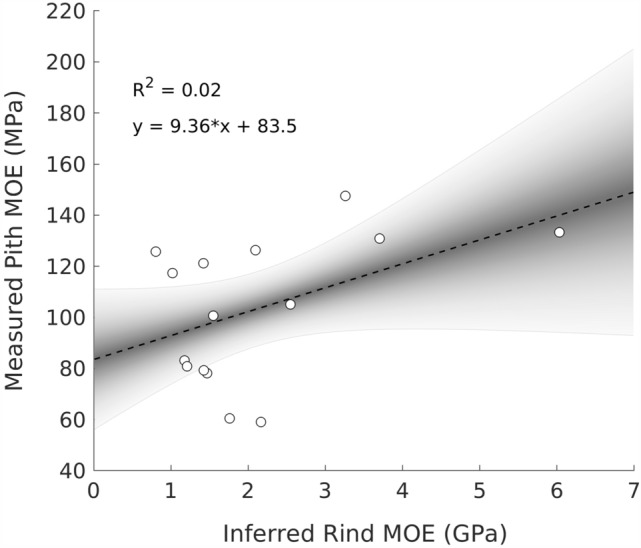
Scatter plot showing the relationship between rind and pith modulus of elasticity (MOE) values. Shading indicates the probability of various relationships between the two properties. The outer edges of the shaded region are 95% confidence levels

A weak positive correlation was found between rind and pith values for both data sets shown in Fig. 2. However, because the directly measured rind and inferred pith values (inner pair in Fig. 2) were found to be less reliable, only the relationship between the measured pith and inferred rind values (outer pair) were investigated further. A scatter plot and linear fit for measured pith and inferred rind values is shown in Fig. 4. The relationship was found to be statistically significant at the 90% confidence level, but not at the 95% confidence level. Given the collected data, the shaded region in Figure 4 illustrates the probability of various possible actual relationships between the two material properties.

### Water content and water loss profiles

**Fig. 5:**
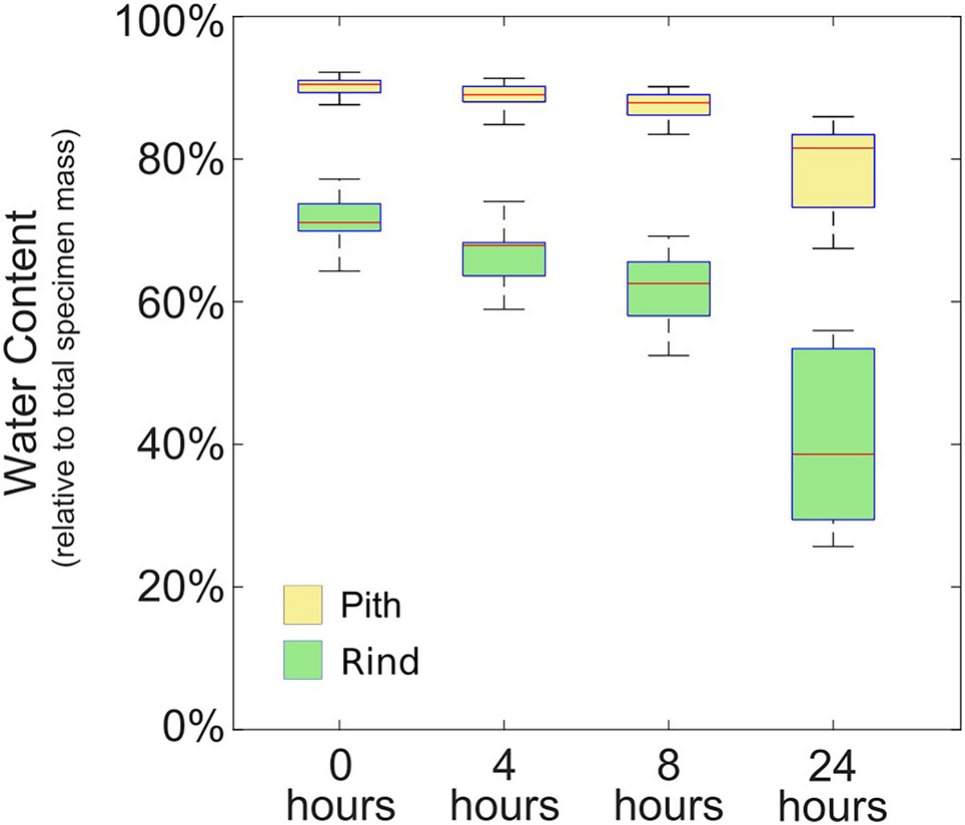
Water content of rind-only and pith-only specimens as functions of time

Rind and pith tissues differed in the amount of initial water content (as a percentage of total specimen mass at each measurement point). Fully hydrated pith tissues were approximately 90% water and 10% dry mass. In contrast, fully hydrated rind tissues ranged from 65 to 75% water (25% to 35% dry mass). As tissues dehydrated, water content of both followed similar patterns. These data are shown graphically in Fig. 5.

**Fig. 6:**
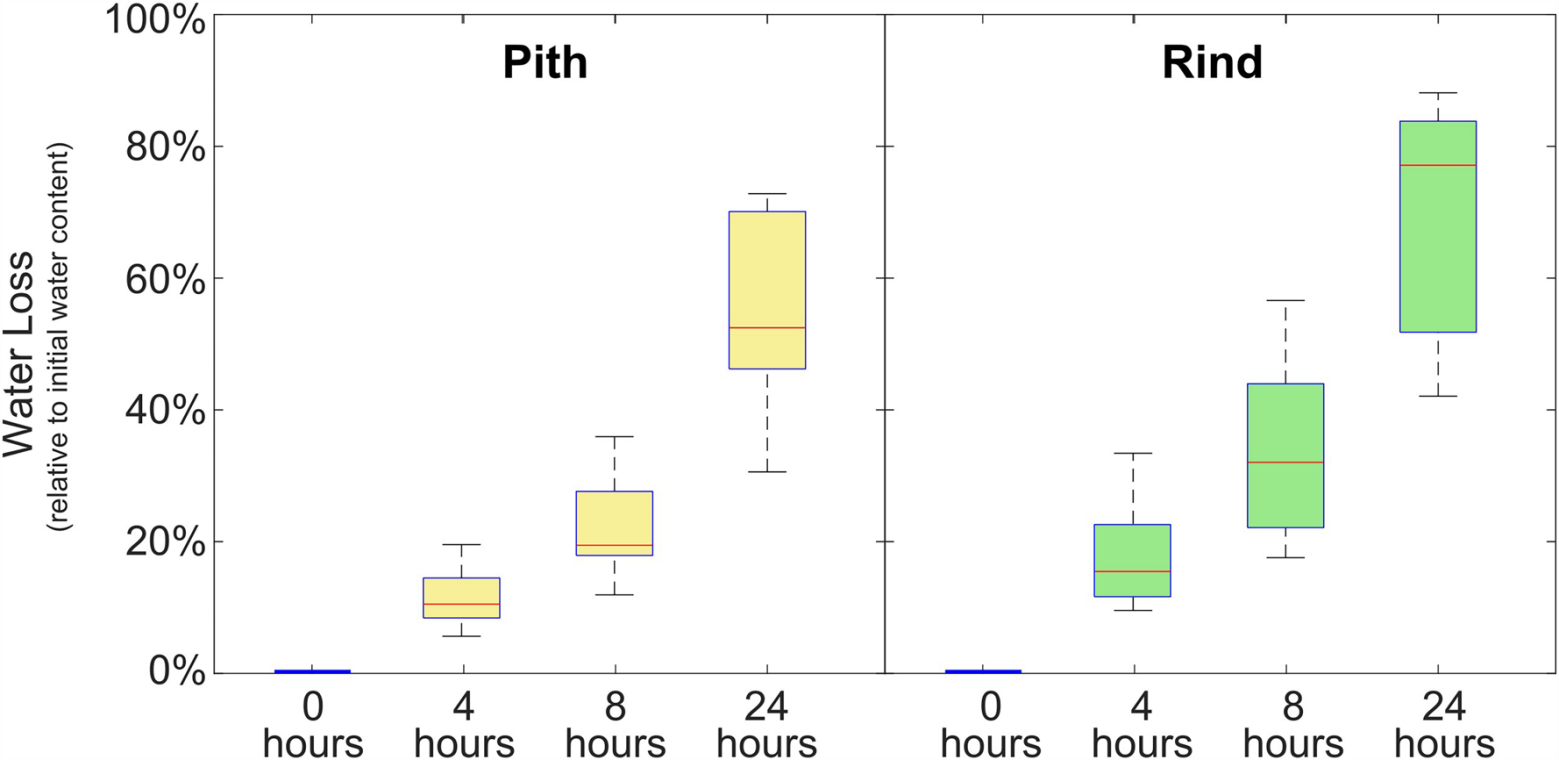
Water loss profiles for pith-only and rind-only specimens

As shown in Fig. 6, more than 50% water loss was observed in most rind and pith samples during the 24-h testing period. At the end of this period, water loss of pith specimens ranged from 30 to 74%. For the rind, water loss at the 24-h mark ranged from 40 to 90%.

### Modulus of elasticity values

**Fig. 7:**
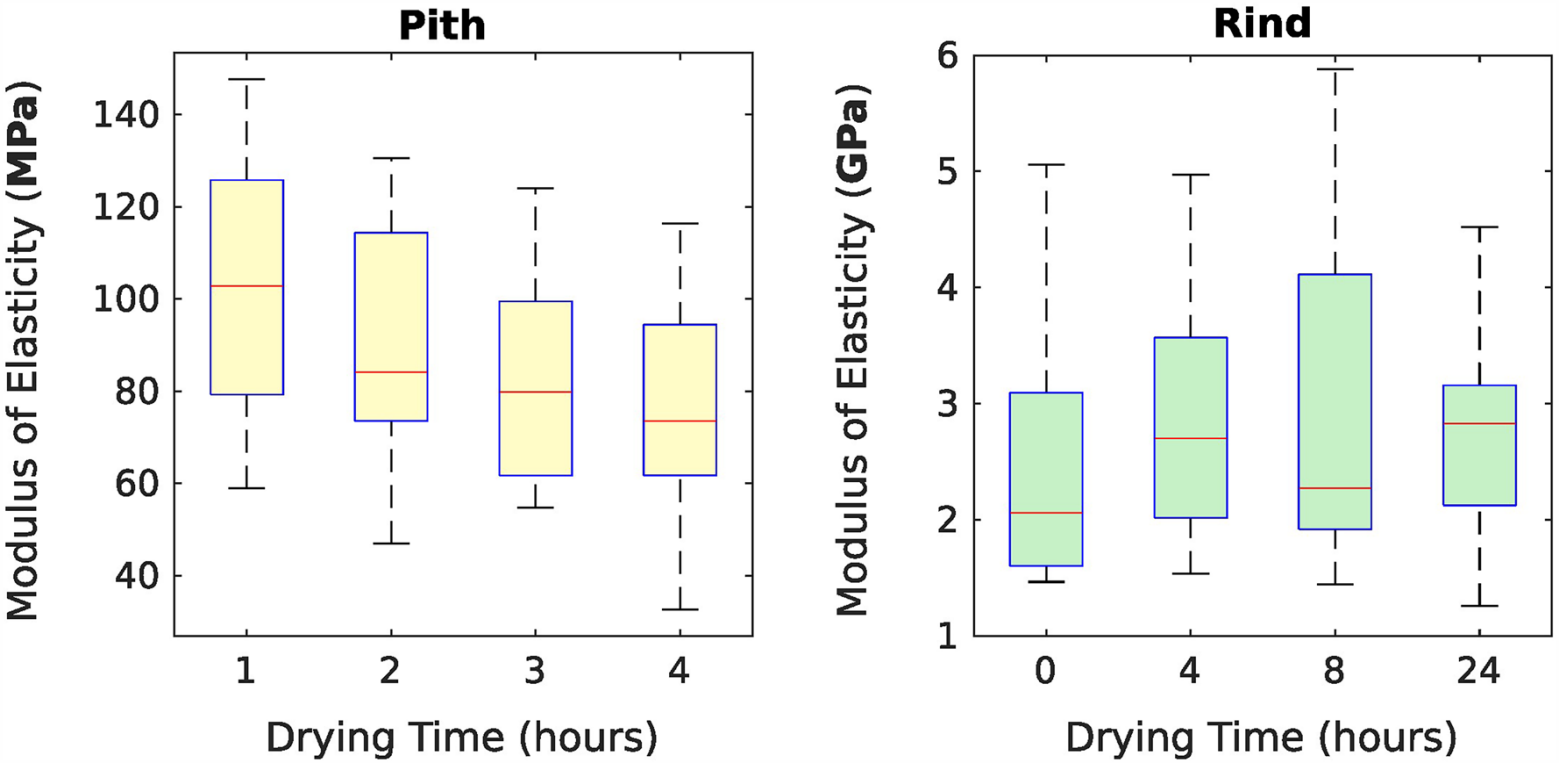
Distributions of modulus of elasticity values for rind and pith tissues at specified time points. Boxes represent the inner quartiles while whiskers represent the 5th and 95th percentile data points

Across all pith specimens (regardless of water content), the median value of the pith modulus was 83.5 MPa. For the rind, the median value was 2.4 GPa. Boxplots showing the distributions of modulus values at drying time points are shown in Fig. 7. As seen in this figure, pith modulus decreased with drying time. There was no clear pattern of change in the rind modulus with drying time.

### Change in stiffness as a function of water loss

**Fig. 8:**
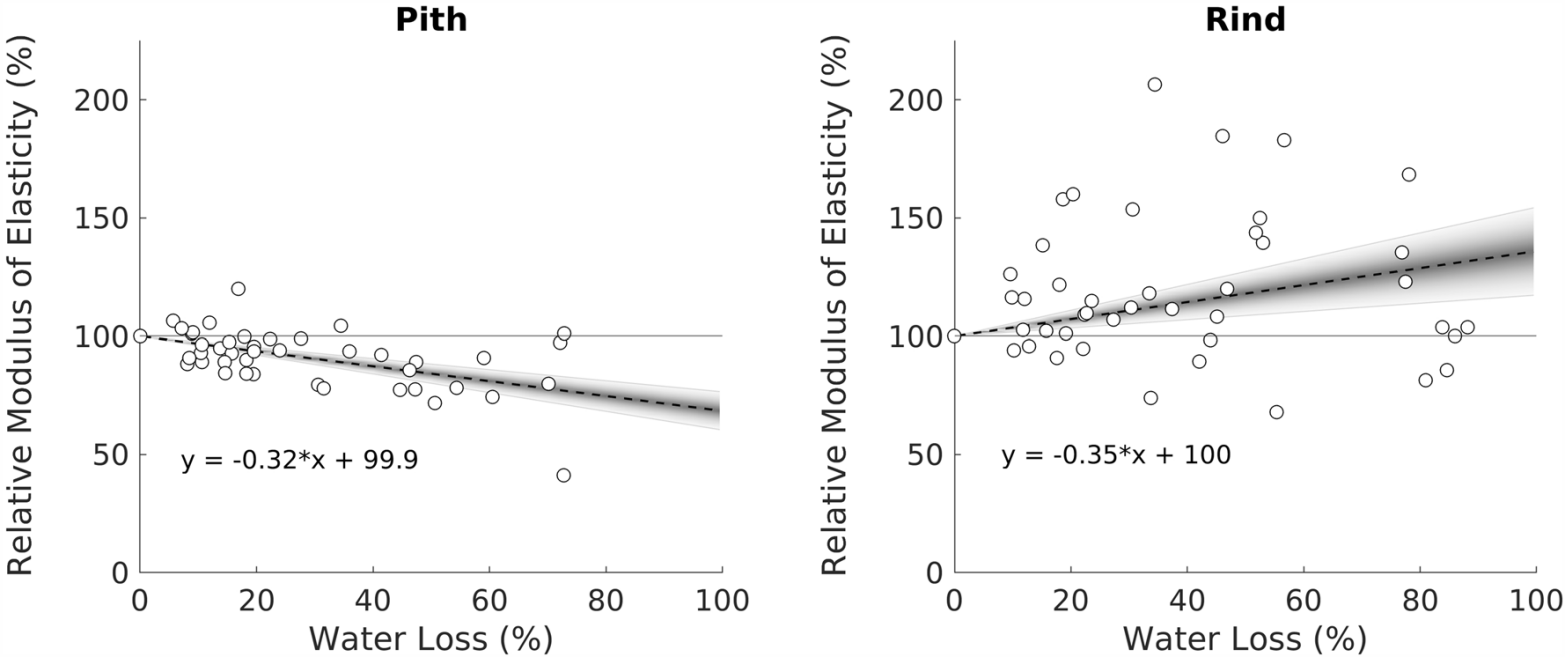
Scatter plots showing the relationship between modulus of elasticity and water loss for pith and rind tissues. Due to the normalization of both axes, the fit lines were required to pass through the point (0, 100). Both fit lines were statistically significant. Shading indicates the 95% confidence levels on the fit lines with the outer boundary corresponding to 95% confidence level

The Water Loss approach provided the clearest depiction of the relationship between water content and modulus of elasticity. Pith samples exhibited a distinct negative relationship between water loss and structural stiffness. As water was removed via evaporation, pith specimens showed a significant decrease in stiffness. This trend is shown in Fig. 8. The regression line of this figure indicates (by extrapolation) that the pith without any water would have roughly^2/3^ the stiffness of the pith when fully turgid.

The rind did not show the same trend. Instead, rind tissues tended to increase in stiffness as water was removed (right-hand panel of Fig. 8). In addition, variation levels for rind specimens was found to be greater than for pith specimens. Overall, it appears that rind tissue was weakly influenced by water content, though the rind results must be interpreted with some caution as the rind-only results are biased by the lateral deflection issue mentioned previously.

## Discussion

### Comparison with previously reported values

One prior study reported the aggregate modulus of elasticity of miscanthus stems as ranging from 0.5 to 6.6 GPa [13]. The specimens in that study exhibited had a relatively low fraction of pith (0 to 67%). In this study, aggregate modulus of elasticity values ranged from 0.2 to 1.4 GPa, with a pith fraction of 78%–89%. Given the higher pith fraction in miscanthus, the values found in this study, it seems reasonable that maize aggregate modulus values would be somewhat lower.

The modulus of dry maize rind tissues have been reported previously as 8–50 GPa [35] and 4–16 GPa (Al-Zube, 2017, 2018). The 8–50 GPa range was obtained using tensile testing of dogbone shaped specimens. This approach is recommended for isotropic materials such as metals [3, 4], but is not appropriate for materials with embedded fibers [5]. This may explain the unusually high upper limit (50 GPa) reported by Zhang et al. As a point of comparison, the modulus of elasticity values reported for dozens of wood species ranged from 4 to 16 GPa [12]. Elsewhere, wood is reported as 6–20 GPa, bamboo at 15–20 GPa, brick, concrete at 25–38 GPa, and magnesium alloys at 42–47 GPa [8]. Given this latter information, it seems highly unlikely that the true value of maize rind modulus exceeds 20 GPa. In this study, the maize rind was always somewhat moist (never fully dry) and modulus values ranged from 0.8 to 6 GPa. This range overlaps with the ranges of dry rind tissues published by Al-Zube (2017, 2018). Given that (a) the rind tissues in this study were wet and (b) the testing method used in this study tended to depress the measured modulus, these values are relatively consistent with prior work.

Previously reported modulus of elasticity for parenchyma tissues vary widely. At the low end, the modulus for potato was reported as 1–19 MPa [21]. Pumpkin parenchyma has been reported at 1–2.5 MPa[18]. The pith of rattan has been reported at 25 MPa [31]. Apple parenchyma has been reported at 10–40 MPa and banana parenchyma at 10–120 MPa [16, 17]. For maize, the pith tissue has been previously reported as 20–190 MPa [36]. However, that study used dogbone-shaped specimens which are not appropriate for fibrous materials [5]. The modulus of elasticity of maize pith reported in this study (33–148 MPa) overlaps significantly with the 20–190 MPa range reported by Zhang et al., but is many times higher than the 1 MPa values reported for potato and pumpkin parenchyma. As a point of practical reference, the modulus of elasticity of human skin has been summarized in a review article as ranging from 4 to 100 MPa [14]

### Relationships between rind and pith tissue properties

When creating computational models, we seek to simulate all the relevant relationships present in reality. Where relationships between mechanical tissue parameters exist, they should be included in computational models. However, such relationships are often not reported in the literature. If important relationships exist without our knowledge, the models we create may be “Frankenstein” models: each component makes sense in isolation, but when the entire model is assembled, it does not adequately represent reality.

In this study, we observed a weak positive relationship between the longitudinal modulus of elasticity of the pith and rind (Fig. 4). A previous study examined the transverse modulus of elasticity of maize pith and rind, but found no relationship [30]. Both studies utilized relatively small sample sizes (*n* = 15 in this study; *n* = 19 in [30]. The weak positive relationship observed in this study was not strong enough to definitively establish a relationship between these two parameters, but the data suggest that a positive relationship may exist. One possible explanation for a relationship between longitudinal modulus but not transverse modulus may lie in the architecture of vascular bundles. The vascular bundles carry the vast majority of the load in both rind and pith tissues. In contrast, vascular bundles carry a much smaller relative amount of load in transverse loading cases. If the stiffness of vascular bundles of rind and pith tissue are related to each other, it stands to reason that there would be a relationship between the modulus of rind tissue and pith tissue, But at this point, further research involving larger sample sizes and multiple measurement modalities is needed to more definitively establish what (if any) relationships exist between the material properties of the rind and pith.

### Influence of water content

The dependencies of pith and rind tissues to water content observed in this study were consistent with those generally reported in the literature for similar tissues of other species: namely a positive relationship between modulus of elasticity and water content for parenchyma (pith), and an inverse relationship for rind tissue. This is explained by the fact that water has two effects in plant tissues: turgidity increases stiffness, but at the same time, wetted cellulose fibers are less stiff than dry cellulose fibers [22]. The net effect depends upon the ratio of stiffening to softening effects.

Water content is significantly higher in parenchyma (pith) tissue than sclerenchyma (rind) tissue, as shown in Fig. 5. In parenchyma tissue, water plays an active structural role by bearing some of the load that is applied to pith-only specimens. This is why turgid parenchyma tissue causes “crispy” carrot sticks and “hard” potatoes. In contrast, lower water content causes flaccid carrots and potatoes. At the same time, water acts to soften cellulose fibers. In high water content tissues, cellulose fibers are softer, but turgidity helps make up the difference.

In contrast, rind tissue seems to derive the vast majority of its stiffness from the cell wall structure since the addition of water acts reduces the stiffness of the rind. In rind tissues like maize, the majority of the load seems to be borne by tightly packed vascular bundles. Water reduces stiffness of this tissue more than it contributes stiffness. The negative relationship between stiffness and moisture observed in this study is consistent with prior studies showing the same result for wood and bamboo [6, 34].

### Testing methodology and limitations

As shown in Fig. 1, modulus of elasticity values of each tissue type were calculated in two different ways: one of which was direct and the other of which was based on inference. This “triangulation” approach is a best practice to confirm that the measurements are reliable [20]. Unfortunately, in this case, these two methods did not provide comparable results (see Fig. 2). There are two reasons for this. First, the direct method requires 4 measured quantities (see Eq. 3), but the indirect method relies upon a total of 8 measurements (see Eqs. 3 and 6). As such, the inference method is more sensitive to error than because it combines all the errors of the direct method plus additional error sources. While this effect may explain the difference in the spread of distributions, it does not explain the fact that inferred values for both rind and pith are shifted upward from the directly measured values.

A second reason for discrepancies is that pith-only and rind-only specimens are not equally amenable to this testing process. The creation of a pith-only specimen requires the removal of all rind tissue from an intact specimen. A sharp razor is used, with cuts chosen to insure that no rind tissue remains on the specimen (more information provided in Additional File 1). In contrast, it is quite difficult to fully remove all pith tissue from an intact specimen to create a rind-only specimen. There are two challenges here: (a) it is difficult to fully remove all pith tissue since the rind becomes increasingly delicate as pith tissue is removed; and (b) any pith tissue that is not removed adversely affects measurements of force, deformation, and tissue area.

The third mechanism that influenced inferred results was the lateral “bowing” of tissues during rind-only compression tests. Lateral deformation is extremely difficult to measure experimentally because the location at which lateral deformation will occur is unpredictable. To gain more insight on this issue, the testing process described above was simulated using finite-element models. Model results mirrored the patterns seen in the measured data in several important ways. First, rind-only models exhibited much greater levels of lateral deformation. Second, lateral deformation reduced the calculated modulus of rind-only specimens. Third, lateral deformation inflated the modulus values of corresponding pith tissues. Additional information on the models created and deformation is provided in Additional File 1. To summarize, lateral bowing introduces an undesirable deformation mode and has the effect of reducing the force/deformation slope of rind-only tests. This causes lower values of rind modulus and consequently, higher values of the inferred modulus values for pith tissues.

With these factors in mind, we can estimate the accuracy of reported values. In order from most to least accurate, these values are (1) directly measured pith values (4 measured quantities, negligible lateral bowing); (2) inferred rind values (8 measured quantities, negligible lateral bowing); (3) directly measured rind values (4 measured quantities which were adversely influenced by factors mentioned above); and (4) inferred pith values (8 measured quantities which were adversely influenced by factors mentioned above). We therefore conclude that the intact and pith-only testing process is reliable, but the rind-only testing process should not be used in future studies.

In addition to the mechanical testing methods listed above, there are other methods for studying the influence of water content on tissue properties. The most popular of these is to assess turgor pressure, either by measuring water potential of the specimens, or by manipulating the turgor pressure through osmotic solutions [18, 21, 26]. These approaches provide additional insight into the mechanics of the pressurized cell membranes, but are also more complex. Future studies should consider using both the mass-based method used in this study alongside the turgor pressure and/or osmotic approach. Combining all three methods in a single study that measures multiple tissue properties with larger sample sizes is needed to fully understand the relationships between mass, turgor pressure, water potential, and the various mechanical tissue properties of plant tissues.

### Alternative testing procedures

In this study, specimens were tested at the turgid state and then dissected. The dissected specimens were then tested as water content was passively decreased through evaporation. This approach allowed for paired rind and pith values only at the initial (turgid) state. As discussed above, rind-only specimens were found to underestimate the true modulus of elasticity of the rind because of lateral bowing. To avoid the problems associated with rind-only specimens, one alternative approach would be to prepare and weigh intact specimens as described above. Intact specimens could then be allowed to air-dry for a period of time, after which they would be weighed, scanned, and tested. Next, the specimen would be dissected to create a pith-only specimen which would be weighed, scanned, and tested. This approach would allow for the direct measurement of pith modulus and water content of the pith. The rind tissue could also be weighed and the rind modulus could be inferred from the test data. Of course, the pith-only specimens could be tested at subsequent intervals. While this would provide additional data, the limiting factor in the alternative approach is that rind modulus could only be obtained when intact and pith-only specimens were tested in conjunction with each other. The advantage of this approach would be that both rind and pith values would be obtained for each specimen at varying water content levels. The disadvantage of this approach is that a larger number of specimens would be required to obtain trends in rind modulus as a function of water content.

## Conclusions

This study provided estimates of the modulus of elasticity of moist maize pith and rind tissues, which are needed to create realistic computational models of maize stems. This study also found that the modulus of elasticity of pith tissues decrease as water loss increases while the modulus of rind tissues tends to increase as water loss increases. This phenomenon is explained by the relative contribution of water to the two tissue types, with water having a significant positive influence on pith modulus, but a lesser, and negative influence on rind tissues. A weak positive relationship was observed between rind and pith tissue. This relationship is hypothesized to be related to the architecture of vascular bundles.

The measurement protocol developed in this study was found to provide useful direct estimates of pith tissues as well as useful indirect estimates of rind tissues. However, the direct measurement of rind tissues is not recommended in future studies because of the potential for measurements to be adversely affected by lateral bowing. As a consequence, the indirect measurement of pith tissues is also not recommended for future studies. This information will support future measurement and modeling research on maize as well as other species having similar architecture (grain sorghum, bioenergy sorghum, miscanthus, sugarcane, etc.).

## Supplementary Information

**Additional file 1.** Additional information about sample preparation and the construction and results of a finite-element model of compression specimens.

## Data Availability

The data set used in this study is available from the authors upon request.

## References

[1] Al-Zube L, Robertson DJ, Edwards JN, Sun W, Cook DD (2017). Measuring the compressive modulus of elasticity of pith-filled plant stems. Plant Methods.

[2] Al-Zube L, Sun W, Robertson D, Cook D (2018). The elastic modulus for maize stems. Plant Methods.

[3] ASTM A370–22. (). Standard Test Methods and Definitions for Mechanical Testing of Steel Products. https://www.astm.org/a0370-22.html. Accessed on Dec 23, 2022.

[4] ASTM E8/E8M-22. (). Standard Test Methods for Tension Testing of Metallic Materials. https://www.astm.org/e0008_e0008m-22.html. Accessed on Dec 23, 2022.

[5] ASTM-D5083. (). Standard Test Method for Tensile Properties of Reinforced Thermosetting Plastics Using Straight-Sided Specimens. ASTM International, West Conshohocken, PA. http://www.astm.org (2017). Accessed on Jan 18, 2018.

[6] Babiak M, Gaff M, Sikora A, Hysek Š (2018). Modulus of elasticity in three-and four-point bending of wood. Compos Struct.

[7] Boresi AP, Schmidt RJ (2002). Advanced mechanics of materials.

[8] Cambridge University Engineering Department. (2003). Materials Data Book. http://www-mdp.eng.cam.ac.uk/web/library/enginfo/cueddatabooks/materials.pdf

[9] Chen YX, Chen J, Zhang YF, Zhou DW (2007). Effect of harvest date on shearing force of maize stems. Livest Sci.

[10] Falk S, Hertz CH, Virgin HI (1958). On the relation between turgor pressure and tissue rigidity. I: experiments on resonance frequency and tissue rigidity. Physiologia Plantarum..

[11] Garber RJ, Olson PJ (1919). A study of the relation of some morphological characters to lodging in cereals 1. Agronomy J.

[12] Green DW, Winandy JE, Kretschmann DE. Mechanical properties of wood. Wood handbook: wood as an engineering material. Forest Products Laboratory. 1999.

[13] Kaack K, Schwarz K-U, Brander PE (2003). Variation in morphology, anatomy and chemistry of stems of Miscanthus genotypes differing in mechanical properties. Ind Crops Prod.

[14] Kalra A, Lowe A, Al-Jumaily AM (2016). Mechanical behaviour of skin: a review. J Mater Sci Eng.

[15] Karam GN, Gibson LJ (1994). Biomimicking of animal quills and plant stems: natural cylindrical shells with foam cores. Mater Sci Eng, C.

[16] Krokida MK, Karathanos VT, Maroulis ZB (2000). Compression analysis of dehydrated agricultural products. Drying Technol.

[17] Krokida MK, Karathanos VT, Maroulis ZB (2000). Effect of osmotic dehydration on viscoelastic properties of apple and banana. Drying Technol.

[18] Mayor L, Cunha RL, Sereno AM (2007). Relation between mechanical properties and structural changes during osmotic dehydration of pumpkin. Food Res Int.

[19] Moulia B, Coutand C, Lenne C (2006). Posture control and skeletal mechanical acclimation in terrestrial plants: Implications for mechanical modeling of plant architecture. Am J Bot.

[20] Nelson N, Stubbs CJ, Larson R, Cook DD (2019). Measurement accuracy and uncertainty in plant biomechanics. J Exp Bot.

[21] Niklas KJ (1988). Dependency of the Tensile Modulus on transverse dimensions, water potential, and cell number of pith parenchyma. Am J Bot.

[22] Niklas KJ (1992). Plant biomechanics: an engineering approach to plant form and function.

[23] Niklas KJ, Moon FC (1988). Flexural stiffness and modulus of elasticity of flower stalks from *Allium*
*Sativum* as measured by multiple resonance frequency-spectra. American J Bot..

[24] Ottesen MA, Larson RA, Stubbs CJ, Cook DD (2022). A parameterised model of maize stem cross-sectional morphology. Biosys Eng.

[25] Prasad J, Gupta CP (1975). Mechanical-properties of Maize stalk as related to harvesting. J Agric Eng Res..

[26] Scanlon MG, Pang CH, Biliaderis CG (1996). The effect of osmotic adjustment on the mechanical properties of potato parenchyma. Food Res Int.

[27] Stubbs CJ, Baban NS, Robertson DJ, Al-Zube L, Cook DD, Geitmann A, Gril J (2018). Bending stress in plant stems: models and assumptions. Plant biomechanics—from structure to function at multiple scales.

[28] Stubbs CJ, Larson R, Cook DD (2020). Mapping spatially distributed material properties in finite element models of plant tissue using computed tomography. Biosys Eng.

[29] Stubbs CJ, Larson R, Cook DD (2022). Maize stalk stiffness and strength are primarily determined by morphological factors. Sci Rep.

[30] Stubbs CJ, Sun W, Cook DD (2019). Measuring the transverse Young’s modulus of maize rind and pith tissues. J Biomech.

[31] Tian G, Shang L, Yang S, Jiang Z (2014). Compression properties of vascular boundles and parenchyma of rattan (*Plectocomia*
*assamica* Griff). Holzforschung.

[32] Tyree MT, Hammel HT (1972). The measurement of the turgor pressure and the water relations of plants by the pressure-bomb technique. J Exp Bot.

[33] Von Forell G, Robertson D, Lee SY, Cook DD (2015). Preventing lodging in bioenergy crops: a biomechanical analysis of maize stalks suggests a new approach. J Exp Bot.

[34] Wang H, Wang H, Li W, Ren D, Yu Y (2013). Effects of moisture content on the mechanical properties of moso bamboo at the macroscopic and cellular levels. BioResources.

[35] Zhang L, Yang Z, Zhang Q, Guo HL (2016). Tensile properties of Maize Stalk Rind. BioResources.

[36] Zhang L, Yang Z, Zhang Q, Zhu X, Hu H (2017). Mechanical behavior of corn stalk pith: an experimental and modeling study. INMATEH-Agric Eng.

