# Supplementary material for: The influence of water content on the longitudinal modulus of elasticity of maize stalk pith and rind tissues
Source: Plant Methods. 2023 Jun 30;19:64. doi: 10.1186/s13007-023-01039-5 (PMC10311759; doi:10.1186/s13007-023-01039-5)
Supplement: Supplementary file 1 — Additional file 1. Additional information about sample preparation and the construction and results of a finite-element model of compression specimens. [file 13007_2023_1039_MOESM1_ESM.pdf]

Supplementary Information

# The Influence of Water Content on the Longitudinal Modulus of Elasticity of Maize Stalk Pith and Rind Tissues

## Additional Details on Sample Preparation and Testing Procedures

*Rind-only Specimens*

Pith tissue was removed from intact specimens by first drilling a 5mm hole down the center of the pith using a common drill bit. Next, the remaining pith was gently removed using a dissection spatula. Care was taken to ensure that the rind did not fracture during this process, and that the drill did not contact the rind. Any samples that were damaged in this process were discarded. Rind-only specimens were subjected to compression tests immediately after removal of the pith.

*Pith-only Specimens*

The rind was removed by separating a small portion of the rind from the pith at the end of the sample with a razor blade and then pulling that strip of rind away from the rest of the sample. This was repeated around the circumference of the sample till the rind was fully removed. If a portion of the rind did not pull away from the pith during the initial removal, the remaining rind would then be carefully shaved off using the razor blade. Pith-only specimens were subjected to compression tests immediately after removal of the rind.

## Compression Testing Equipment

Compression testing was performed with a TestResources 240-1125-41(Test Resources, Shakopee, MN) universal test machine equipped with two spherical rotating platens (Al-Zube et al., 2017). Two self-leveling laser levels were used to ensure the proper alignment of the specimen and the platens. The laser levels were placed such that they projected perpendicularly to each other and projected down the center of the platen. The samples were then placed between the two platens such that both lasers ran parallel and down the center of the sample.

## Exploring Lateral Deformation using Finite-Element Models

Ideally, axial compression tests will produce only axial deformation and no other types of deformation. As explained in the body of this paper, lateral deformation (bowing) during compression tests will lead to erroneous results. Based on our experience in performing these types of tests, we suspected that lateral bowing of the rind-only specimens caused a reduction in the calculated values of rind modulus and a corresponding increase in the pith modulus. Finite-element models were created to explore this possibility. Because the focus of the paper is on the testing methodology and results, these models are only used help explain discrepancies observed between testing methods (main paper, Figure 3).

Finite-element models were constructed from CT scan data of maize stalks. The CT scans had a spatial resolution of 92 μm/voxel. Like the physical specimens, models were extracted from the center of each internode and were 6cm in length. The models were created using ABAQUS, a commercial finite-element program. Boundary conditions matched those of the self-aligning platens used in the physical tests. A displacement of 0.1 mm was applied to the top face of intact and rind-only models.

Finite-element simulations revealed that lateral bowing of rind-only specimens was 10-15 times greater than lateral bowing of pith-only and intact specimens. As seen in the experiments, lateral bowing decreased the apparent stiffness of the rind-only specimens, leading to decreased modulus values for the rind tissue and inflated modulus values for pith tissues. The figure below helps to illustrate this phenomenon.

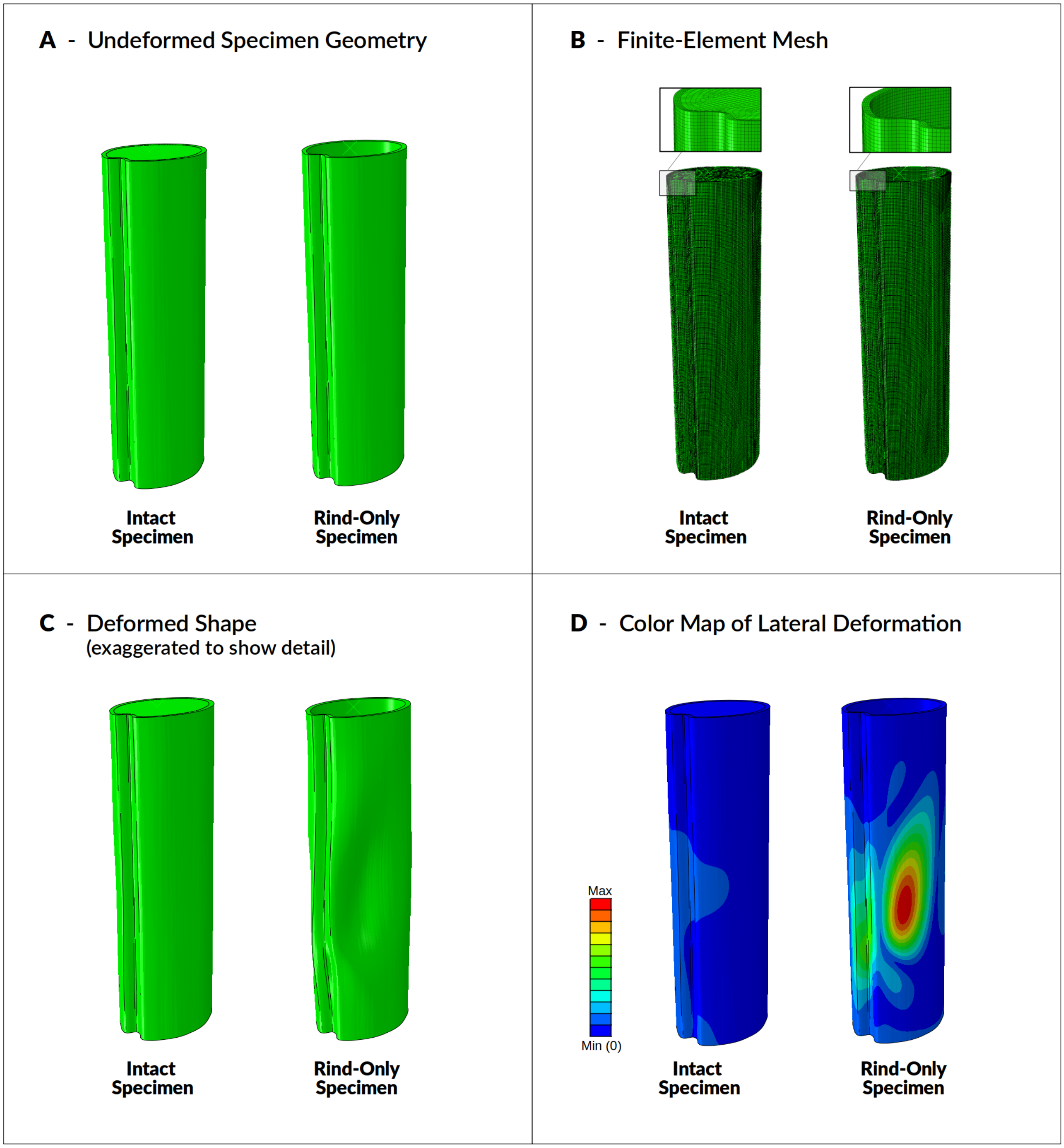


***Supplementary Figure 1:*** *Comparisons between several aspects of intact and rind-only maize stalk internode specimens. Scaling is identical for each pair of images. Note that deformed shapes have been exaggerated to aid in visualization. The specimens with color maps depict only lateral deformation of the stalk (i.e., the magnitude of all deformation perpendicular to the compression axis). The measurement of lateral deformation is challenging because both intact and rind-only specimens have large regions of very low transverse deformation and because the regions of maximum transverse deformation cannot be determined before testing.*
